# Potentially burdensome care at the end-of-life for cancer decedents: a retrospective population-wide study

**DOI:** 10.1186/s12904-024-01358-x

**Published:** 2024-02-02

**Authors:** Rebecca J Mitchell, Geoffrey P Delaney, Gaston Arnolda, Winston Liauw, Jane L Phillips, Reidar P Lystad, Reema Harrison, Jeffrey Braithwaite

**Affiliations:** 1 Australian Institute of Health Innovation, Faculty of Medicine, Health and Human Sciences, Macquarie University, Level 6, 75 Talavera Road, Sydney, NSW 2109 Australia; 2 Maridulu Budyari Gumal - Sydney Partnership for Health, Education, Research and Enterprise (SPHERE), UNSW, Sydney, Australia; 3 Cancer Therapy Centre, Liverpool Hospital, Sydney, Australia; 4 Collaboration for Cancer Outcomes Research and Evaluation, South-Western Sydney Clinical School, UNSW, Sydney, Australia; 5 University of New South Wales School of Clinical Medicine, Sydney, Australia; 6 Cancer Care Centre, St George Hospital, Kogarah, Australia; 7 Faculty of Health, School of Nursing, QUT, Brisbane, Australia

**Keywords:** End-of-life-care, Health care utilisation, Palliative care, Cancer

## Abstract

**Background:**

Variation persists in the quality of end-of-life-care (EOLC) for people with cancer. This study aims to describe the characteristics of, and examine factors associated with, indicators of potentially burdensome care provided in hospital, and use of hospital services in the last 12 months of life for people who had a death from cancer.

**Method:**

A population-based retrospective cohort study of people aged ≥ 20 years who died with a cancer-related cause of death during 2014–2019 in New South Wales, Australia using linked hospital, cancer registry and mortality records. Ten indicators of potentially burdensome care were examined. Multinominal logistic regression examined predictors of a composite measure of potentially burdensome care, consisting of > 1 ED presentation or > 1 hospital admission or ≥ 1 ICU admission within 30 days of death, or died in acute care.

**Results:**

Of the 80,005 cancer-related deaths, 86.9% were hospitalised in the 12 months prior to death. Fifteen percent had > 1 ED presentation, 9.9% had > 1 hospital admission, 8.6% spent ≥ 14 days in hospital, 3.6% had ≥ 1 intensive care unit admission, and 1.2% received mechanical ventilation on ≥ 1 occasion in the last 30 days of life. Seventeen percent died in acute care. The potentially burdensome care composite measure identified 20.0% had 1 indicator, and 10.9% had ≥ 2 indicators of potentially burdensome care. Compared to having no indicators of potentially burdensome care, people who smoked, lived in rural areas, were most socially economically disadvantaged, and had their last admission in a private hospital were more likely to experience potentially burdensome care. Older people (≥ 55 years), females, people with 1 or ≥ 2 Charlson comorbidities, people with neurological cancers, and people who died in 2018–2019 were less likely to experience potentially burdensome care. Compared to people with head and neck cancer, people with all cancer types (except breast and neurological) were more likely to experience ≥ 2 indicators of potentially burdensome care versus none.

**Conclusion:**

This study shows the challenge of delivering health services at end-of-life. Opportunities to address potentially burdensome EOLC could involve taking a person-centric approach to integrate oncology and palliative care around individual needs and preferences.

**Supplementary Information:**

The online version contains supplementary material available at 10.1186/s12904-024-01358-x.

## Introduction

Cancer is one of the leading causes of death internationally [1], and in Australia, around 151,000 people are newly diagnosed with cancer each year, with an estimated 1.3 million cancer-related hospital admissions [2]. Cancer-related morbidity and mortality is predicted to increase due to an ageing and increasing population [3–5]. This will create challenges for the provision of cancer management, including end-of-life-care (EOLC) [3]. Due to differing approaches to EOLC management, EOLC can be complex to navigate and also to evaluate [6]. Availability of hospital, palliative or hospice services, resources, level of community support, and both patient and clinical characteristics can all influence the quality of EOLC [7, 8].

Towards the end-of-life (EOL), there may be an increase in hospital-based care to actively address potentially reversible conditions (e.g. infections) or at points where the person’s prognosis may be uncertain. Some people experiencing cancer, however, may undergo care that could be viewed as ‘potentially burdensome’ in their last few weeks of life (e.g. multiple hospital or intensive care unit (ICU) admissions, or intravenous (IV) chemotherapy in last 2 weeks of life) that could negatively impact on their quality of life [6, 9, 10]. This sort of potentially burdensome care aimed at prolonging life can sometimes prevail over more comfort-based care [9, 10], and can reduce the amount of time that people spend in their preferred place at their EOL, which is often their home [11]. However, the majority of cancer patients die in health facilities [12]. Previous research identified that 18% of people with cancer (up to 65% if deaths in acute care are included) in Switzerland [7], 22% in Canada [13], 30% in the United States (US) [14], and 34% in the Netherlands [9] experienced at least one indicator of potentially burdensome care at the EOL.

Variation in the quality of EOLC is notable and remains an area where healthcare improvements could be explored [6, 15]. Potentially burdensome EOLC can be costly [16], and not add benefit to a cancer patient’s EOL quality [9]. Indicators of potentially burdensome care towards the EOL for people with advanced cancer have been recommended in prior literature and can be derived from population-based administrative data collections [7, 14, 17, 18] which can provide a cost-effective method of identifying potential variation in EOLC, but have not been extensively examined in Australia. Examination of hospital-based EOLC quality indicators in Australia could pinpoint variation in care delivered at the EOL for people with cancer and indicate opportunities for EOLC quality improvement measures. It may also identify opportunities where EOLC could be enhanced, with the potential to reduce any unnecessary hospital visits, and enhance 24-hour home-based palliative care and home care services. This study aims to describe the characteristics of, and examine factors associated with, indicators of potentially burdensome care provided in hospital and use of hospital services in the last 12 months of life for people who had a death from cancer in New South Wales (NSW), Australia.

## Method

This is a retrospective cohort study of people who had a death from cancer in NSW, Australia, during 1 January 2014 to 31 December 2019. Mortality data was linked to hospital and cancer registry records for 365 days prior to the date of death.

### Data sources and linkage

Mortality data were obtained from the NSW Registry of Births, Deaths and Marriages and the cause of death unit record file (COD-URF) and included date of death and underlying and up to 20 antecedent causes of death. Cause of death was classified using the International Classification of Diseases, 10th Revision (ICD-10). The NSW Cancer Registry records notifications of people with cancer in NSW (except for non-melanoma skin cancer) and includes information on demographics, diagnosis date, cancer type and degree of spread, place of death, and cause of death. NSW Cancer Registry records were provided from 1972 to 2019 to identify diagnosis date and previous history of cancer.

Hospital records were obtained for non-admitted patient occasions of service (i.e. outpatients), emergency department (ED) presentations, and hospital admissions in NSW. Non-admitted patient records included clinical or therapeutic services provided by NSW Health that warrant a note regarding the service being included in the client’s medical record. The non-admitted patient dataset was available as a calendar year from 1 January 2016 and includes public hospitals and information on client demographics, type of service, type of service contact (e.g. in-person, videoconference) and service provider. Non-admitted patient data where there was no client contact were excluded from service counts.

ED presentations to public hospitals in NSW included information on arrival and departure times, visit types and separation type. Hospital admissions were to all public and private hospitals, and information available included principal and additional diagnoses, clinical procedures, and separation type (e.g. hospital transfer, death). Diagnoses were classified using the International Classification of Diseases, 10th Revision Australian-modification (ICD-10-AM). Country of birth was identified using the Standard Australian Classification of Countries [19] in the hospital records and was categorised as Australia and other countries.

The data sources were linked by the Centre for Health Record Linkage (CHeReL) using probabilistic linkage. Upper and lower probability cut-offs for a link were 0.75 and 0.25 and record groups with probabilities between the cut-offs were clerically reviewed.

### Case inclusion criteria

Cases included individuals aged ≥ 20 years with a cancer-related cause of death (ICD-10: C00-C96, D45, D46, D47.1, D47.3-D47.5) in their underlying cause of death in the COD-URF during 2014–2019. Cancer type was identified using cause of death records (Supplementary Table S1). There were *n* = 15,020 deaths excluded from analysis as the underlying cause of death was not recorded as cancer, but cancer was identified in any of 20 antecedent causes of death. Also excluded were *n* = 1,340 deaths from non-melanoma skin cancer (ICD-10: C44) and individuals that died within 30 days of diagnosis (*n* = 9,609). The hospital service use of individuals who received a non-admitted patient service or who had an ED presentation or a hospital admission and separation within 365 days of their date of death were examined. There were *n* = 2,392 (3.0%) individuals who had a hospital separation during the 365 days before death, but their hospital admission was pre-365 days before death, and these individuals were included in the analysis of hospital service use.

### Residents of aged care and place of death

Patients who were living in residential age care during their last hospital admission before their death were identified using any one or a combination of data from hospital records, including separation mode, source of referral, and financial class (i.e. visit payment). Death in acute care was identified using admission and separation dates, date of death and separation mode (i.e., died) in hospital admission records. Death in the ED was identified using separation mode. Where the individual had not used hospital services, location of death was identified using place of death recorded in the NSW Cancer Registry.

### Palliative or hospice care

Palliative or hospice care was identified using any one or a combination of data items in hospital admission records that indicated palliative or hospice care (i.e. episode of care type, service-related group, unit type on admission, peer-group, facility type, separation mode) or an additional diagnosis in up to 50 diagnosis codes of palliative care (ICD-10-AM: Z51.5) [20].

### ICU admissions and mechanical ventilation

Hospital admission records identify hours in an intensive care unit (ICU) and hours were categorised as ICU admission (Y/N). Likewise, hours on mechanical ventilation are recorded in hospitalisation records and categorised as mechanical ventilation (Y/N).

### EOLC indicators

Eleven indicators of potentially burdensome care at EOL were identified from the literature and expert opinion (Table 1). One identified EOLC indicator of ‘new’ chemotherapy episodes was not able to be identified using the available data. In line with earlier studies, a composite measure of potentially burdensome care was defined using four of the ten indicators as at least one occurrence of either: (i) > 1 ED presentation within 30 days of death; or (ii) > 1 hospital admission within 30 days of death; or (iii) ≥ 1 ICU admission within 30 days of death; or (iv) died in acute care hospital – all excluding palliative/hospice care [6, 17]. Patients were categorised as experiencing none, 1 indicator, or ≥ 2 indicators of potentially burdensome care [6, 17].

**Table 1:** EOLC indicators of potentially burdensome care and potentially adequate symptom management

Indicator	Definition	Source
Potentially burdensome care at EOL in hospital		
A	> 1 ED visit in last 30 days of life	[6, 7, 13, 14, 24, 26]
B	> 1 hospitalisation in last 30 days of life (excluding palliative or hospice care)	[6, 7, 13, 24, 26]
C	Admission to ICU in last 30 days of life	[6, 7, 13, 14, 18, 24–28]
D	Place of death at EOL was in acute care (excluding palliative or hospice care)	[7, 14, 25]
E	Spending ≥ 14 days in hospital in last 30 days of life (excluding palliative or hospice care)	[14]
F	≥ 3 hospitalisations in last 90 days of life (excluding palliative or hospice care)	[7]
G	Mechanical ventilation in last 30 days of life	[18, 27]
H	Radiotherapy in last 30 days of life	[36]
I	New IV chemotherapy commencing in last 30 days of life	[7, 14, 18, 27, 36]
J	Last dose IV chemotherapy in last 14 days of life	[6, 7, 13, 14, 24, 26]
K	Last dose IV chemotherapy in last 7 days of life	^1^

^1^ Personal communication, Liauw W, 22 April 2022

### Identification of comorbidities

The Charlson Comorbidity Index was used to identify comorbidities using up to 50 diagnosis classifications in hospitalisation records [21]. A one-year lookback was applied from the date of death to identify comorbidities in the hospital admission data (i.e. to 1 January 2013). Charlson comorbidities, excluding malignancies, were categorised as nil, 1 and ≥ 2 comorbidities. Comorbid conditions related to depression (ICD-10-AM: F20.4, F31.3, F31.4, F31.5, F32, F33, F34.1, F41.2, F43.2), anxiety-related disorders (ICD-10-AM: F40-F48), alcohol misuse and dependence (ICD-10-AM: F10, Y90, Y91, Z50.2, Z71.4, Z72.1), drug-related dependence (ICD-10-AM: F11-F16, F19, Z50.3, Z71.5, Z72.2), and tobacco use (ICD-10-AM: F17.0-F17.9, P04.2, T65.2, Z58.7, Z71.6, Z72.0, Z81.2, Z86.43) were also identified using hospital records.

### Socio-economic status and geographic location

An indicator of socio-economic disadvantage was assigned using the index of relative socioeconomic disadvantage [22] and Statistical Area Level 2 (SA2) of residence in hospital or NSW Cancer Registry records. The values were partitioned into quintiles from most (i.e. 1) to least disadvantaged (i.e. 5). The quintiles are derived from Australia’s population census using information including education, employment, occupation and income. The Australian Statistical Geographical Standard Remoteness Area [23] and SA2 of residence in hospital records or NSW Cancer Registry records was used to derive the five remoteness categories, based on distance to service centres. These categories were collapsed into: urban (i.e. major cities) and rural (i.e. inner regional, outer regional, remote, and very remote).

### Chemotherapy and radiotherapy

Chemotherapy administered in hospital was identified using a principal diagnosis of cancer (ICD-10-AM: C00-C96, D45, D46, D47.1, D47.3-D47.5) and the principal or up to 50 additional procedure block codes indicating ‘administration of pharmacotherapy’ (1920) or ‘other procedures related to pharmacotherapy’ (1922) [2] or an Australian-refined diagnosis related group (AR-DRG) of chemotherapy (R63Z). IV chemotherapy or radiotherapy administered via outpatients was identified using the non-admitted patient data collection service type and service classification of chemotherapy or radiotherapy, respectively.

### Data management and analysis

Data were analysed using SAS 9.4 (SAS Institute, Cary NC). All hospital episodes of care related to the same event were linked to form a period of care. Descriptive analysis was used to describe the number of non-admitted patient services, ED presentations and hospital admissions in the last 12 months prior to death. Chi-square tests of independence, one-way ANOVA or Kruskal-Wallis Test, as appropriate, were used to examine the characteristics of individuals who received none, 1 or ≥ 2 indicators of potentially burdensome care at EOL.

Multivariable, multinominal logistic regression was used to examine predictors of indicators of potentially burdensome care. Variables included in the model were identified from the literature [6, 7, 13, 14, 18, 24–28] and available in the data, and included age at death, sex, cancer type, cancer degree of spread, history of other cancer, count of Charlson comorbidities (excluding malignancies) (i.e. 0, 1, ≥ 2 comorbidities), depression, anxiety, tobacco use, alcohol or drug dependence, survival duration from date of diagnosis during the study timeframe to date of death (i.e. 31–89 days, ≥ 90 days to < 180 days, ≥ 180 days), urban/rural residence, socioeconomic status, year of death, and public/private hospital at last admission prior to date of death. A backwards stepwise regression sequentially eliminated factors from the model that did not significantly contribute to risk of burdensome care at a significance level of *p* < 0.05 that were screened at ≤ 0.2 in univariable analysis [29] (i.e. cancer degree of spread, depression, and drug-related dependence). Odds ratios (OR) and 95% confidence intervals (CIs) were calculated.

Predictors associated with each of the 11 indicators of potentially burdensome EOLC were also examined individually using multivariable logistic regression (Supplementary Figure S1a and S1b). Variables included in the models were the same as for the multinominal logistic regression and a backwards stepwise regression sequentially eliminated factors at *p* < 0.05, that were screened a significance level at ≤ 0.2 in univariable analysis [29]. ORs and 95% CIs were calculated.

## Results

**Fig. 1:**
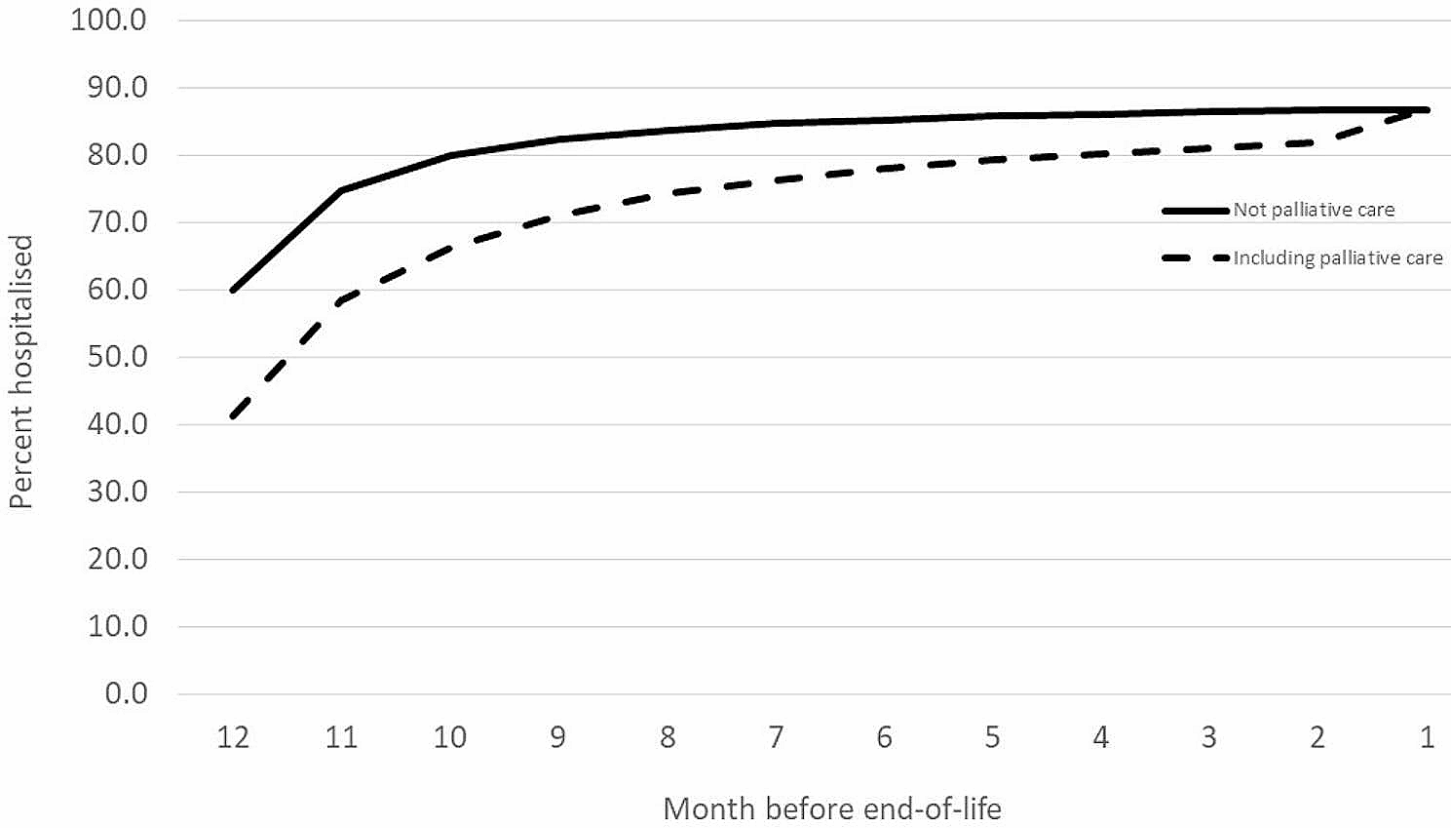
Proportion of decedents hospitalised by month in last 12 months of life

There were 80,005 cancer deaths in NSW identified in the COD-URF during the seven year period. Of these, 3,013 (3.8%) deaths were either not reported or did not link to a record in the NSW Cancer Registry. Of the 80,005 decedents, 86.9% were hospitalised in the 12 months prior to death. The proportion of people hospitalised were lowest in the 12 months before death (41.3% including palliative care and 60.0% not including palliative care) and highest in the month before death (86.9% including palliative care and 86.7% excluding palliative care) (Fig. 1). The most common cancer types were lung (18.1%), digestive organs (excluding colorectal) (17.0%), colorectal (11.8%), and blood and lymphatic system (11.4%).

For the decedents in their last 30 days of life, 14.8% had > 1 ED presentation, 9.9% had > 1 hospital admission, 3.6% had ≥ 1 ICU admission, and 1.2% had ≥ 1 mechanical ventilation – all excluding palliative and hospice care. 17% of people died in acute care (and 52.7% died in hospital including non-acute palliative and hospice care), 8.6% had ≥ 14 days spent in hospital in the last 30 days of life, and 18.2% had ≥ 3 hospital admissions in the last 90 days of life. Demographic, cancer and clinical characteristics varied by indicator type (Supplementary Tables S2 and S3). In the year before death, there was an increase in the mean number of hospital admissions excluding palliative care (from 0.8 to 3.1 admissions per month), hospital admissions including palliative care (0.8 to 4.6) and ED presentations (0.7 to 3.1) (Supplementary Figure S2).

There were 53,523 decedents during 2016 to 2019 where non-admitted patient data was available. Of these, 3.5% and 7.3% had a dose of chemotherapy in their last 7 days and 14 days of life, respectively, and 5.7% received radiotherapy in their last 30 days of life. Demographic characteristics of decedents varied by indicator type (Supplementary Table S4). Radiotherapy within the last 30 days of life was most commonly provided to people with lung cancer (33.0%). IV chemotherapy within the last 7 and 14 days of life was most commonly administered to people with lung cancer (21.3% and 21.0%, respectively), cancers of the blood and lymphatic system (17.5% and 18.1%, respectively), and cancers of the digestive organs, excluding colorectal cancer (16.6% and 16.3%, respectively) (Supplementary Table S5). In the last 12 months of life, the mean number of non-admitted patient occasions of service (after excluding non-client contacts) increased from 11.7 to 49.9 per month (Supplementary Figure S3).

The potentially burdensome care composite measure identified that 69.2% of decedents did not have any of the four indicators of potentially burdensome care, 20.0% had one indicator, and 10.9% had ≥ 2 indicators. People who had a higher proportion of 1 or ≥ 2 indicators of potentially burdensome care compared to none, were younger (20–54 year) (9.7% and 11.9% vs. 7.2%), male (59.6% and 62.6% vs. 54.8%), had no comorbidities (37.7% and 43.3% vs. 30.7%), were smokers (50.2% and 58.2% vs. 40.4%), and died in hospital (68.3% and 73.3% vs. 42.3%), respectively (Tables 2 and 3).

**Table 2:** Demographic characteristics of the cancer decedents by potentially burdensome care composite index

	None (*n* = 55,343; 69.2%)		1 indicator (*n* = 15,979; 20.0%)		≥ 2 indicators (*n* = 8,683; 10.9%)		*p*-value
	*n*	%	*n*	%	*n*	%	
**Mean age at death** (SD)	74.5	(12.7)	71.8	(12.8)	69.6	(12.5)	< 0.0001
**Age group**							
20–54	3,974	7.2	1,556	9.7	1,031	11.9	< 0.0001
55–64	7,579	13.7	2,586	16.2	1,627	18.7	
65–74	13,503	24.4	4,589	28.7	2,750	31.7	
75–84	16,883	30.5	4,651	29.1	2,392	27.6	
≥ 85	13,404	24.2	2,597	16.3	883	10.2	
**Sex**							
Male	30,348	54.8	9,524	59.6	5,434	62.6	< 0.0001
Female	24,995	45.2	6,455	40.4	3,249	37.4	
**Country of birth**							
Australia	36,909	66.7	11,219	70.2	6,172	71.1	< 0.0001
Other country/not known	18,434	33.3	4,760	29.8	2,511	28.9	
**Number of Charlson comorbidities, excluding malignancy** ^1^							
Nil	16,966	30.7	6,030	37.7	3,755	43.3	< 0.0001
1 comorbidity	19,808	35.8	6,212	38.9	3,382	39.0	
≥ 2 comorbidities	9,036	16.3	2,810	17.6	1,546	17.8	
Not known (no hospital admission)	9,533	17.2	927	5.8	0	-	
**Other comorbidities**							
Depression (yes) ^1^	314	0.6	93	0.6	40	0.5	0.05
Anxiety-related disorder (yes) ^1^	1,335	2.4	382	2.4	200	2.3	0.001
Tobacco use (yes) ^1^	22,378	40.4	8,026	50.2	4,925	56.7	< 0.0001
Alcohol misuse and dependence (yes) ^1^	792	1.4	258	1.6	146	1.7	1.0
Drug-related dependence (yes) ^1^	268	0.5	114	0.7	72	0.8	0.007
**Geographical location of residence** ^1^							
Urban	31,934	57.7	9,293	58.2	5,329	61.4	< 0.0001
Rural	13,569	24.5	5,582	34.9	3,212	37.0	
Not known	9840	17.8	1,102	6.9	142	1.6	
**Socio-economic status** ^1^							
Most disadvantaged	10,329	18.7	3,694	23.1	2,009	23.1	< 0.0001
2	10,922	19.7	4,127	25.8	2,410	27.8	
3	9,058	16.4	2,778	17.4	1,657	19.1	
4	6,419	11.6	1,759	11.0	993	11.4	
Least disadvantaged	8,774	15.9	2,519	15.8	1,473	17.0	
Not known	9,841	17.8	1,102	6.9	141	1.6	
**Resident of aged care** (yes) ^1^	4,044	7.3	390	2.4	82	0.9	< 0.0001
**Year of death**							
2014	8,875	16.0	2,623	16.4	1,422	16.4	0.17
2015	9,299	16.8	2,771	17.3	1,492	17.2	
2016	9,036	16.3	2,588	16.2	1,441	16.6	
2017	9,202	16.6	2,576	16.1	1,492	17.2	
2018	9,252	16.7	2,671	16.7	1,386	16.0	
2019	9,679	17.5	2,750	17.2	1,450	16.7	

^1^ Not known excluded from chi-square test of independence; including *n* = 9533 (17.2%) for none, *n* = 927 (5.8% for 1 indicator and nil for ≥ 2 indicators for depression, anxiety, tobacco and alcohol use

**Table 3:** Cancer and clinical characteristics of the cancer decedents by potentially burdensome care composite index

	None (*n* = 55,343; 69.2%)		1 indicator (*n* = 15,979; 20.0%)		≥ 2 indicators (*n* = 8,683; 10.9%)		*p*-value
	*n*	%	*n*	%	*n*	%	
**Age at diagnosis**, median (SD)	73.0	(5.6)	70.0	(13.3)	68.0	(12.9)	< 0.0001
**Time from diagnosis to death** (years), median (SD)	2.2	(7.5)	2.0	(7.1)	1.9	(6.7)	< 0.0001
**History of cancer** (yes)	14,126	26.6	4,204	27.2	2,372	28.3	0.003
**Survival duration** (days) ^1^							
31–89	5,292	9.6	1,684	10.5	926	10.7	< 0.0001
≥ 90 days to < 180	4,970	9.0	1,514	9.5	844	9.7	
≥ 180	42,887	77.5	12,251	76.7	6,624	76.3	
Not known	2,194	4.0	530	3.3	289	3.3	
**Cancer type**							
*Head and neck*	1,544	2.8	435	2.7	180	2.1	< 0.0001
*Digestive organs, excl. colorectal*	9,302	16.8	2,829	17.7	1,469	16.9	
*Colorectal*	6,759	12.2	1,788	11.2	929	10.7	
*Lung*	9,800	17.7	3,022	18.9	1,676	19.3	
*Melanoma of the skin*	1,980	3.6	515	3.2	303	3.5	
*Mesothelial and soft tissue*	1,413	2.6	395	2.5	211	2.4	
*Breast*	4,129	7.5	1,048	6.6	502	5.8	
*Female genital organs, excl. ovarian*	1,277	2.3	291	1.8	127	1.5	
*Ovarian*	1,217	2.2	311	2.0	174	2.0	
*Prostate*	4,372	7.9	1,050	6.6	459	5.3	
*Kidney*	1,086	2.0	301	1.9	169	2.0	
*Bladder*	1,253	2.3	347	2.2	173	2.0	
*Neurological*	1,903	3.4	346	2.2	118	1.4	
*Blood and lymphatic system*	5,339	9.7	2,168	13.6	1,578	18.2	
*Unknown primary site*	2,861	5.2	761	4.8	418	4.8	
*Other cancers and other ill-defined sites*	1,108	2.0	372	2.3	197	2.3	
**Degree of cancer spread**							
In-situ/localised	11,486	20.8	2,986	18.7	1,491	17.2	< 0.0001
Regionalised	10,983	19.5	3,241	20.3	1,650	19.0	
Metastatic	17,019	30.8	5,004	31.3	2,789	32.1	
Not known	15,855	28.7	4,748	29.7	2,753	31.7	
**Place of death**							
Home	8,722	15.8	1,075	6.7	317	3.7	< 0.0001
Hospice	1,265	2.3	126	0.8	55	0.6	
Hospital	23,425	42.3	10,906	68.3	6,363	73.3	
Residential aged care	9,163	16.6	367	2.3	119	1.4	
Not known	12,768	23.1	3,505	21.9	1,829	21.1	

^1^ Not known excluded from the chi-square test of independence

**Fig. 2:**
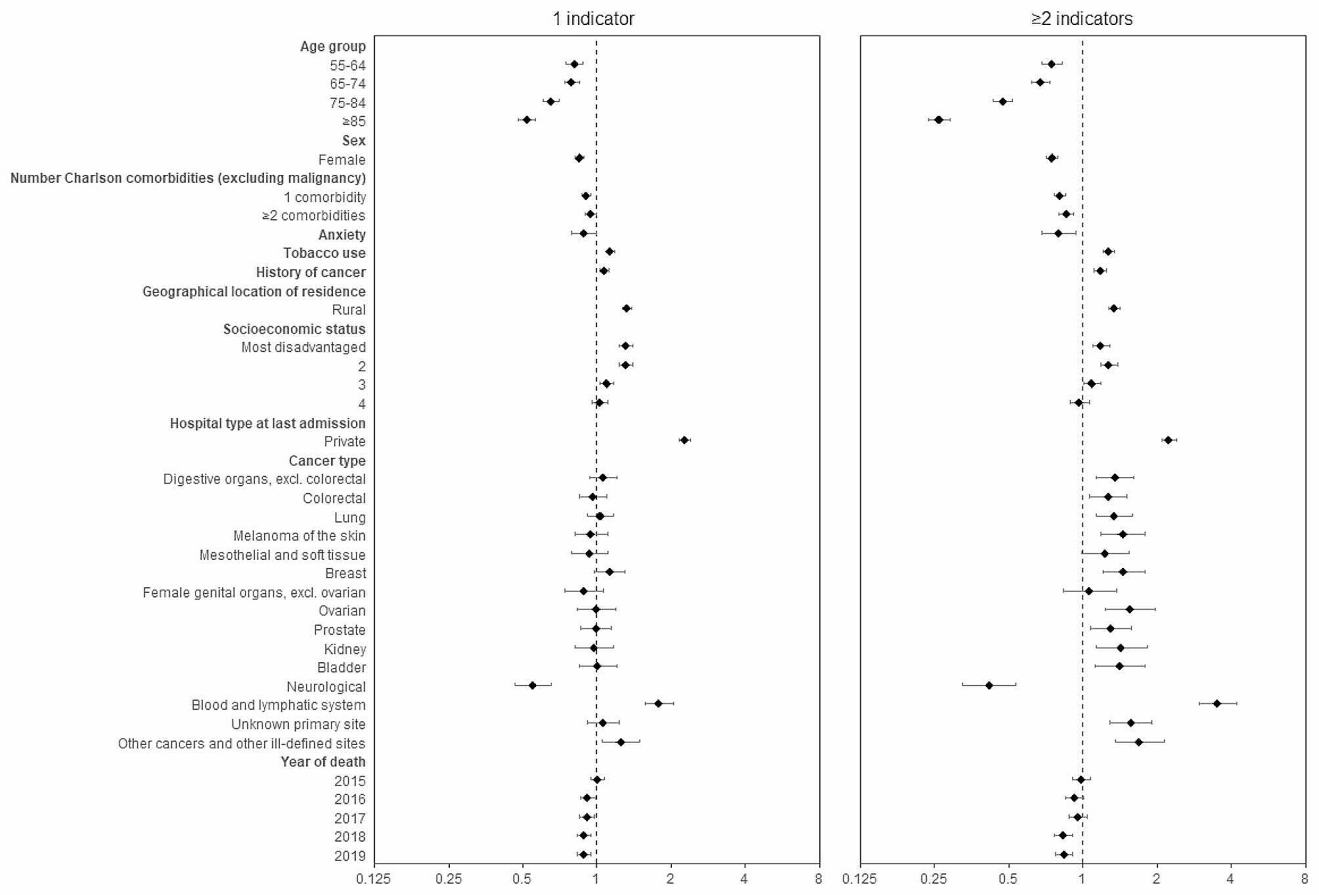
Multinominal model of characteristics associated with potentially burdensome care at the end of life^1^ ^1^ Reference categories were: Nil composite indicators of potentially burdensome care, 20–54 years, males, nil comorbidities, no history of cancer, urban location, most advantaged socioeconomic status, public hospital, head and neck cancer, and death in 2014

Compared to having no composite indicators of potentially burdensome care, people who smoked, lived in rural locations, were in the most disadvantaged socio-economic group, and had their last admission before death in a private hospital were more likely to experience 1 or ≥ 2 indicators of potentially burdensome care than referent groups. The odds of experiencing ≥ 2 indicators of potentially burdensome care compared to none was lower for people with head and neck cancer, compared to people with each of the other cancer types except for breast and neurological cancers. The following sub-groups were less likely to experience both 1 or ≥ 2 indicators of potentially burdensome care, compared to none: older people (≥ 55 years), females, people with 1 or ≥ 2 Charlson comorbidities, people with neurological cancers, and people who died in 2018 or 2019 compared to referent groups (Fig. 2).

## Discussion

This study used linked mortality, hospital, and cancer registry records to examine indicators of potentially burdensome EOLC for people whose principal cause of death was cancer in the last 12 months of life. The proportion of decedents who used hospital services increased in the last months of life. The results indicate that between 1.2% and 18.2% of decedents met at least one of 11 indicators of potentially burdensome care in the last 12 months of life. Several characteristics, including people who smoked, lived in rural locations, were most socioeconomically disadvantaged, had certain cancer types, and who had their last admission in a private hospital, were associated with people experiencing 1 or ≥ 2 indicators of potentially burdensome care compared to none.

Potentially burdensome EOLC can result in poor quality of life [30] and signal challenges in integrated care coordination [7]. In the last 30 days of life, the current study identified that 14.8% of decedents had > 1 ED presentation. The proportion of ED presentations identified was similar to Alberta, Canada (12.5%) [6], the Netherlands (12% who had > 2 ED visits) [9], Taiwan (18.3%), and Ontario, Canada, for people with advanced pancreatic cancer (18.5%) [24], but is seven times higher than Switzerland (2.1%) [7], and lower than Belgium (33.8%) [10]. Predictors associated with each of the seven indicators of potentially burdensome EOLC were generally similar, except for > 1 ED visit in last 30 days of life, where people with a lower socioeconomic status had a higher likelihood of visiting the ED and were less likely to have their last hospital admission in a private hospital, both likely reflecting reduced access to other care options and lack of private health insurance, respectively.

The current study identified that for people with cancer in the last 30 days of life, 9.9% had > 1 hospital admission, 8.6% spent ≥ 14 days in acute care, 3.6% had ≥ 1 ICU admission, and 1.2% had ≥ 1 mechanical ventilation. A range of factors including differing practices, resource availability, and patient demographic and clinical factors [31–33] are likely responsible for differences in EOLC hospital service use for people with cancer reported in other countries in the last 30 days of life (Table 4). It is also possible that the use of ED and/or hospitalisation may be being used as a substitute for community-based palliative care [34], which would have implications for service design. In particular, private hospitals may be acting as a de-facto hospice, especially in regional areas. However, it should be acknowledged that lower rates of ED presentations or hospital admissions may not represent ‘good’ EOLC [32], but do potentially signal the challenges people living with advanced cancer at home experience accessing timely community based 24-hour cancer, palliative and/or primary care.

**Table 4:** Potentially burdensome EOLC in last 30 days of life by country and indicator

Country and timeframe	Cancer	> 1 ED presentation	> 1 hospital admission (%)	≥ 1 hospital admission (%)	> 2 hospital admissions (%)	≥ 1 ICU admission (%)	> 14 days in hospital (%)	≥ 1 mechanical ventilation (%)
New South Wales, 2014–2021 (current study)	All	14.8	9.9	-	-	3.6	8.6	1.2
Alberta, 2006–2009 [6];	Colorectal	-	9.5	-	-	2.2	-	-
Austria, 2012–2016 [36]	All	-	-	-	-	8.6	-	-
Belgium, 2012 [10]	Advanced pancreatic	-	-	62.0	-	-	-	-
Ontario, 2005–2010 [24]	All	-	8.3	-	-	4.3	-	-
Netherlands, 2017 [9]	All	-	-	-	9	6	8	-
Switzerland, 2014 [7]	All	-	6	-	-	6.9	-	-
Taiwan, 2000–2016 [37]	All	-	14.2	-	-	11.4	44	28.5
United Kingdom, 2010–2017 [31]	All	37.6	49.4	-	-	2.4	-	-

In the current study, the mean number of ED presentations, hospital admissions and non-admitted patient occasions of service increased in the last 12 months, as has been demonstrated elsewhere [31]. Fewer ED presentations near EOL has been flagged as a signal of the strength of integration of palliative and oncology services within acute care [35]. However, a rise in use of hospital services towards the EOL is not unique to cancer [34]. It is also possible that some of the hospital service use within the 12-month EOL period were not related to cancer treatment but were for other causes.

In the current study, 17% of people died in acute care. This proportion compares similarly to Netherlands (20%) [9], but is less than half the proportion reported in other countries, including Alberta, Canada, (50%) [6], Austria (53%) [36], Switzerland (56%) [7], and Taiwan (63%) [37]. Differences in the number of acute care deaths between countries could reflect the availability of 24 h home-based palliative care services or hospice beds [14], good symptom and pain management, adequate family support, and patient preferences regarding the location of their death and their families ability to support their wishes [11, 33].

Four percent of decedents had a dose of chemotherapy in their last 7 days of life and 7.3% had a dose in their last 14 days of life in the current study. Similar proportions of people with cancer received their last dose of chemotherapy within their last 7 and 14 days of life in Belgium (4% and 8.6%, respectively) [10], within their last 14 days of life in Switzerland (7%) [7] and in Ontario (7.1%) [24]. Almost double the proportion of people with cancer (15.7%) received chemotherapy within their last 14 days of life in the US [14], while a systematic review identified that between 1 and 19% of patients received chemotherapy in their last 14 days of life [32]. Chemotherapy provided near EOL may be of limited benefit and signal overuse [26, 38]. However, patient preferences also should be considered; previous research identified that patients who preferred life-extending care were likely to receive chemotherapy within 2 weeks of death compared to patients who preferred comfort-orientated care [39].

Within the current study, 5.7% of decedents received radiotherapy in their last 30 days of life. In comparison, in Austria, 1.7% of people with cancer received radiotherapy in 30 days before death [36]. While radiotherapy can assist in pain management for advanced cancer, its provision close to EOL is not likely to be efficacious [38].

There were 20% and 11% of decedents, respectively, in the current study that had 1 or ≥ 2 indicators of potentially burdensome EOLC. In the Netherlands, around one-third of cancer decedents experienced potentially burdensome EOLC in the 30 days before EOL [9]. In Switzerland, 23.8% of decedents had one and 40.4% had ≥ 2 indicators of potentially burdensome care [7]. The proportional variation in the presence of potentially burdensome indicators of EOL between countries may be attributable to differences in cohort inclusion criteria, the type of composite indicators included, and/or availability of palliative care services [40].

The current study identified that people who smoked, lived in rural locations, who were most socioeconomically disadvantaged were more likely to experience burdensome care, while older people (≥ 55 years), females, people with comorbidities, were less likely to experience indicators of potentially burdensome care. Similarly, previous research also identified that people with comorbidities [6, 7], people who lived in rural locations [6], older people and females were less likely to receive potentially burdensome treatment at EOL [6, 10, 14].

Use of palliative care has the potential to reduce potentially burdensome EOLC and pain [7, 24]. Within Australia, an estimated 42% of hospital-based palliative care was for patients with a principal diagnosis of cancer [41]. The provision of palliative care in the last 30 days of life has been associated with people with cancer being five times less likely to experience potentially burdensome EOLC [9]. Any treatment provided at EOL should have the potential to improve symptoms/comfort, be consistent with patient and family preferences [14], involve shared decision-making and consider cost-efficient resource utilisation by avoiding high-cost acute resources [39, 42].

There is scope for future research to examine predictors of potentially burdensome care by cancer type, geographic location, and also to consider indicators of the use of specialist oncology services and pharmaceutical use, such as opioid prescriptions, towards the EOL [10]. The timing of the use of palliative and hospice care, examining group-based trajectories of hospital service use at EOL, and further comparison between jurisdictions may aid to identify differing or similar care practices for sub-population groups. Predictive computer models, using machine learning, to better identify patients near their EOL using mortality prediction modelling may aid decision making of clinicians as well [43].

The strengths of the study include that it was population-based and included multiple linked data collections that enabled capture of hospital service use. The study considered all cancer types and all ages ≥ 20 years. As to limitations, the EOLC indicators provide an indication of the quality of healthcare services rather than the quality of care provided to individuals, therefore the absence of an EOLC indicator does not ensure the provision of good quality EOLC, as a person with cancer who died at home may not have had access to good community or home-based palliative care support [6]. Alternatively, the presence of an EOLC indicator does not mean a patient received poor quality care, as some acute care may be clinically warranted [9]. More granular data about the reason for admission and procedures administered would be able to provide insight as to whether treatment could be viewed as not burdensome to the individual. Further, it is not possible to predict exactly when a person with cancer may die, and some people may be admitted for curative treatment and die unexpectedly.

Whether the radiotherapy was single or multi-fraction external beam radiotherapy could not be identified – for treatment of bone metastases. NSW residents who lived near the borders of NSW whose death was recorded in NSW may have received hospital-based treatment interstate, and no information was available on their health service use. Under-enumeration of care is particularly likely for people with cancer who received chemotherapy and radiotherapy interstate, as northern NSW residents often travel to Queensland, southern NSW residents to Victoria, and south-western NSW residents to South Australia to receive treatment [44]. These patients represent a small proportion of all NSW cancer patients. In the current study, no information was available regarding other health service use (e.g. primary care, palliative care and home care services), pharmaceutical prescriptions or advance care planning. There is likely under-enumeration of people with cancer who received palliative or hospice care and the identification of aged care residents in hospital records. Within hospital records, only comorbidities that had an impact on patient care are recorded, which is likely to result in under-enumeration of chronic health conditions. However, by using a one year look back period, better estimates of the prevalence of health conditions were likely able to be obtained in the current study [45]. Information on primary care was not available for this study, which may have included information on comorbidities. Hospital data validity was not able to be assessed. It is acknowledged that the potential burdensome indicators of EOLC focus on care provided in acute care, thus an individual who presented to ED only (and who was not admitted to hospital), would only have the potential to experience one potentially burdensome indicator of EOLC and would not have been exposed to other indicators considered for admitted patients only.

## Conclusion

This study identified that in last month of life, some cancer decedents experienced potentially burdensome treatments. It signals the challenge of health service delivery at the EOL and can provide a reference baseline for future work examining EOLC for people with cancer. There are likely opportunities to address any potentially burdensome EOLC by taking a person-centric approach and integrating oncology and palliative care around individual needs and preferences.

### Electronic supplementary material

Below is the link to the electronic supplementary material.

**Supplementary Material 1: Table S1**: Identification of cancer type. **Table S2**: Demographic characteristics of the cancer decedents by potentially burdensome ED and hospital admission care indicators. **Table S3**: Cancer and clinical characteristics of the cancer decedents by potentially burdensome ED and hospital admission care indicators. **Table S4**: Demographic characteristics of the cancer decedents by potentially burdensome chemotherapy or radiotherapy indicators during 2016-2019. **Table S5**: Cancer and clinical characteristics of the cancer decedents by potentially burdensome chemotherapy or radiotherapy indicators during 2016-2019. **Figure S1a**: Predictors of characteristics associated with potentially burdensome care at the end of life by indicator type, 2014-2019. **Figure S1b**: Predictors of characteristics associated with potentially burdensome care at the end of life by indicator type, 2014-2019. **Figure S2**: Mean number of hospital admissions (a) and ED presentations (b) by month in the last 12 months of life, 2014-2019. **Figure S3**: Mean number of non-admitted patient occasions of service1 by month in the last 12 months of life, 2016-2019

## Data Availability

The data that support the findings of this study are available from the NSW Ministry of Health and the NSW Cancer Institute. Restrictions apply to the availability of these data, which were used under licence for the current study, so are not publicly available.

## References

[1] International Agency for Research on Cancer. Global cancer observatory. 2022 15/03/2022; Available from: https://gco.iarc.fr/

[2] Australian Institute of Health and Welfare (AIHW). *Cancer in Australia 2021*, in *Cancer series no. 133*. Australian Institute of Health and Welfare: Canberra; 2021.

[3] Pilleron S, Soto-Perez‐de‐Celis E, Vignat J, Ferlay J, Soerjomataram I, Bray F, Sarfati D (2021). Estimated global cancer incidence in the oldest adults in 2018 and projections to 2050. Int J Cancer.

[4] Araghi M, Soerjomataram I, Jenkins M, Brierley J, Morris E, Bray F, Arnold M (2019). Global trends in colorectal cancer mortality: projections to the year 2035. Int J Cancer.

[5] Luo G, Zhang Y, Etxeberria J, Arnold M, Cai X, Hao Y, Zou H (2023). Projections of lung cancer incidence by 2035 in 40 countries worldwide: Population-based study. JMIR Public Health and Surveillance.

[6] Hu W, Yasui Y, White J, Winget M (2014). Aggressiveness of end-of-life care for patients with colorectal cancer in Alberta, Canada: 2006–2009. J Pain Symptom Manage.

[7] Bähler C, Signorell A, Blozik E, Reich O (2018). Intensity of treatment in Swiss cancer patients at the end-of-life. Cancer Manage Res.

[8] Earle CC (2005). Evaluating claims-based indicators of the intensity of end-of-life cancer care. Int J Qual Health Care.

[9] Boddaert MS, Pereira C, Adema J, Vissers KC, van der Linden YM, Raijmakers NJ, Fransen HP (2022). Inappropriate end-of-life cancer care in a generalist and specialist palliative care model: a nationwide retrospective population-based observational study. BMJ Supportive & Palliative Care.

[10] De Schreye R, Smets T, Annemans L, Deliens L, Gielen B, De Gendt C, Cohen J (2017). Applying quality indicators for administrative databases to evaluate end-of-life care for cancer patients in Belgium. Health Aff.

[11] Nilsson J, Blomberg C, Holgersson G, Carlsson T, Bergqvist M, Bergström S (2017). End-of‐life care: where do cancer patients want to die? A systematic review. Asia‐Pacific J Clin Oncol.

[12] Swerissen H, Duckett S (2014). Dying well.

[13] Ho TH, Barbera L, Saskin R, Lu H, Neville BA, Earle CC (2011). Trends in the aggressiveness of end-of-life cancer care in the universal health care system of Ontario, Canada. J Clin Oncol.

[14] Earle CC, Neville BA, Landrum MB, Ayanian JZ, Block SD, Weeks JC (2004). Trends in the aggressiveness of cancer care near the end of life. J Clin Oncol.

[15] Gusmano MK, Rodwin VG, Weisz D, Cottenet J, Quantin C (2021). Variation in end-of-life care and hospital palliative care among hospitals and local authorities: a preliminary contribution of big data. Palliat Med.

[16] Reeve R, Srasuebkul P, Langton JM, Haas M, Viney R, Pearson S-A (2018). Health care use and costs at the end of life: a comparison of elderly Australian decedents with and without a cancer history. BMC Palliat Care.

[17] Earle CC, Park ER, Lai B, Weeks JC, Ayanian JZ, Block S (2003). Identifying potential indicators of the quality of end-of-life cancer care from administrative data. J Clin Oncol.

[18] Ersek M (2017). Association between aggressive care and bereaved families’ evaluation of end-of-life care for veterans with non-small cell lung cancer who died in Veterans affairs facilities. Cancer.

[19] Australian Bureau of Statistics (ABS). *Standard Australian classification of countries. Cat no: 1269.0* 2016 21/02/2023].

[20] Australian Institute of Health and Welfare (2022). Palliative care outcomes.

[21] Quan H (2011). Updating and validating the Charlson Comorbidity Index and score for risk adjustment in hospital discharge abstracts using data from 6 countries. Am J Epidemiol.

[22] ABS. 2033.0.55.001 - Census of population and housing: socio-economic indexes for areas (SEIFA). 2011; Available from: http://www.abs.gov.au/ausstats/abs@.nsf/mf/2033.0.55.001/

[23] Australian Bureau of Statistics. Australian statistical geographical standard remoteness area. Australian Bureau of Statistics: Canberra; 2011.

[24] Jang RW, Krzyzanowska MK, Zimmermann C, Taback N, Alibhai SM (2015). Palliative care and the aggressiveness of end-of-life care in patients with advanced pancreatic cancer. J Natl Cancer Inst.

[25] Ní Chróinín D (2018). Health-services utilisation amongst older persons during the last year of life: a population-based study. BMC Geriatr.

[26] Earle CC, Landrum MB, Souza JM, Neville BA, Weeks JC, Ayanian JZ (2008). Aggressiveness of cancer care near the end of life: is it a quality-of-care issue?. J Clin Oncol.

[27] Chiang J-K, Kao Y-H (2017). Predictors of high healthcare costs in elderly patients with liver cancer in end-of-life: a longitudinal population-based study. BMC Cancer.

[28] Luta X, Diernberger K, Bowden J, Droney J, Hall P, Marti J. Intensity of care in cancer patients in the last year of life: a retrospective data linkage study. Br J Cancer. 2022:1–8. 10.1038/s41416-022-01828-0 PMC9092325 35545681

[29] Hosmer D, Lemeshow S (2000). Applied logistic regression. Second edition.

[30] Oselin K, Pisarev H, Ilau K, Kiivet R-A (2021). Intensity of end-of-life health care and mortality after systemic anti-cancer treatment in patients with advanced lung cancer. BMC Cancer.

[31] Luta X, Diernberger K, Bowden J, Droney J, Hall P, Marti J (2022). Intensity of care in cancer patients in the last year of life: a retrospective data linkage study. Br J Cancer.

[32] Langton JM, Blanch B, Drew AK, Haas M, Ingham JM, Pearson S-A (2014). Retrospective studies of end-of-life resource utilization and costs in cancer care using health administrative data: a systematic review. Palliat Med.

[33] Bekelman JE (2016). Comparison of site of death, health care utilization, and hospital expenditures for patients dying with cancer in 7 developed countries. JAMA.

[34] Rosenwax LK, McNamara BA, Murray K, McCabe RJ, Aoun SM, Currow DC (2011). Hospital and emergency department use in the last year of life: a baseline for future modifications to end-of-life care. Med J Aust.

[35] Hui D (2015). Indicators of integration of oncology and palliative care programs: an international consensus. Ann Oncol.

[36] Robausch M, Grössmann N, Wild C (2021). Cancer care near the end-of‐life in Austria: a retrospective data analysis. Eur J Cancer Care.

[37] Tang S, Wu S-C, Hung Y-N, Huang E-W, Chen J-S, Liu T-W (2009). Trends in quality of end-of-life care for Taiwanese cancer patients who died in 2000–2006. Ann Oncol.

[38] Ong WL (2017). Patterns of health services utilization in the last two weeks of life among cancer patients: experience in an Australian academic cancer center. Asia-Pac J Clin Oncol.

[39] Wright AA (2016). Family perspectives on aggressive cancer care near the end of life. JAMA.

[40] Soares LGL, Gomes RV, Palma A, Japiassu AM (2020). Quality indicators of end-of-life care among privately insured people with cancer in Brazil. Am J Hospice Palliat Medicine®.

[41] Australian Institute of Health and Welfare (2023). Hospitals - admitted patients.

[42] Luta X (2021). Evidence on the economic value of end-of-life and palliative care interventions: a narrative review of reviews. BMC Palliat Care.

[43] Vu E (2023). Applications of machine learning in Palliative Care: a systematic review. Cancers.

[44] Gabriel G, Barton M, Delaney GP (2015). The effect of travel distance on radiotherapy utilization in NSW and ACT. Radiother Oncol.

[45] Preen DB, CD’Arcy JH, Spilsbury K, Semmens JB, Brameld KJ (2006). Length of comorbidity lookback period affected regression model performance of administrative health data. J Clin Epidemiol.

