# Supplementary material for: Potentially burdensome care at the end-of-life for cancer decedents: a retrospective population-wide study
Source: BMC Palliat Care. 2024 Feb 2;23:32. doi: 10.1186/s12904-024-01358-x (PMC10835903; doi:10.1186/s12904-024-01358-x)
Supplement: Supplementary file 1 — Supplementary Material 1: Table S1: Identification of cancer type. Table S2: Demographic characteristics of the cancer decedents by potentially burdensome ED and hospital admission care indicators. Table S3: Cancer and clinical characteristics of the cancer decedents by potentially burdensome ED and hospital admission care indicators. Table S4: Demographic characteristics of the cancer decedents by potentially burdensome chemotherapy or radiotherapy indicators during 2016-2019. Table S5: Cancer and clinical characteristics of the cancer decedents by potentially burdensome chemotherapy or radiotherapy indicators during 2016-2019. Figure S1a: Predictors of characteristics associated with potentially burdensome care at the end of life by indicator type, 2014-2019. Figure S1b: Predictors of characteristics associated with potentially burdensome care at the end of life by indicator type, 2014-2019. Figure S2: Mean number of hospital admissions (a) and ED presentations (b) by month in the last 12 months of life, 2014-2019. Figure S3: Mean number of non-admitted patient occasions of service1 by month in the last 12 months of life, 2016-2019 [file 12904_2024_1358_MOESM1_ESM.docx]

**Supplementary Tables and Figures**

**Table S1: Identification of cancer type**

| Cancer type | ICD-10 classification |
| --- | --- |
| All cancers | C00-C96, D45, D46, D47.1, D47.3-D47.5 |
| *Head and neck* | C00-C14, C30-C32 |
| *Digestive organs, excl. colorectal* | C15-C17, C21-C25, C26.1-C26.9 |
| *Colorectal* | C18-C20, C26.0 |
| *Lung* | C33-C34 |
| *Melanoma of the skin* | C43 |
| *Mesothelial and soft tissue* | C45-C49 |
| *Breast* | C50 |
| *Female genital organs, excl. ovarian* | C51-C55, C57-C58 |
| *Ovarian* | C56 |
| *Prostate* | C61 |
| *Kidney* | C64 |
| *Bladder* | C67 |
| *Neurological* | C71, C72.8, C72.9 |
| *Blood and lymphatic system* | C81, C86, C88, C90, C96, D45-D46, D47.1, D47.3, D47.5 |
| *Unknown primary site* | C80 |
| *Other cancers^1^ and other ill-defined sites* | C30-C32, C37-C39, C40-C41, C60, C62-C63, C65-C66, C68-C70, C72.0, C72.7, C75.1, C75.3, C73-C74, C75.0, C75.4, C75.9, C76, |

Source: Appendix B. AIHW Cancer in Australia. 2021 Canberra: AIHW.

^1^Other category includes: nasal cavity, middle ear and sinuses, larynx, other thoracic and respiratory organs, bone, other urinary organs, eye, other central nervous system, thyroid and other endocrine glands, and male genital organs, excluding prostate.

**Table S2: Demographic characteristics of the cancer decedents by potentially burdensome ED and hospital admission care indicators**

|  | **>1 ED visit in last 30 days**  (n=11,844; 14.8%) | | **>1 admission in last 30 days**^1^ (n=7,884; 9.9%) | | **≥1 admission to ICU in last 30 days**  (n=2,883; 3.6%) | | **≥1 mechanical ventilation in last 30 days**  (n=969; 1.2%) | | **Place of death acute care**^1^ (n=13,552; 16.9%) | | **≥14 days in hospital in last 30 days**^1^  (n=6,898; 8.6%) | | **≥3 admissions in last 90 days**^1^  (n=14,560; 18.2%) | |
| --- | --- | --- | --- | --- | --- | --- | --- | --- | --- | --- | --- | --- | --- | --- |
|  | n | % | n | % | n | % | n | % | n | % | n | % | n | % |
| **Mean age at death** (SD) | 70.6 | (12.7) | 69.5 | (12.6) | 67.2 | (12.5) | 66.2 | (12.9) | 71.8 | (12.5) | 72.3 | (12.2) | 69.4 | (12.4) |
| **Age group** |  |  |  |  |  |  |  |  |  |  |  |  |  |  |
| 20-54 | 1,327 | 11.2 | 9.9 | 11.9 | 441 | 15.3 | 168 | 17.3 | 1.260 | 9.3 | 556 | 8.1 | 1,705 | 11.7 |
| 55-64 | 2,111 | 17.8 | 1,534 | 19.5 | 595 | 20.6 | 197 | 20.3 | 2,126 | 15.7 | 1,083 | 15.7 | 2,832 | 19.5 |
| 65-74 | 3,467 | 29.3 | 2,471 | 31.3 | 987 | 34.2 | 343 | 35.4 | 4,069 | 30.0 | 2,078 | 30.1 | 4,754 | 32.7 |
| 75-84 | 3,375 | 28.5 | 2,107 | 26.7 | 704 | 24.4 | 211 | 21.8 | 4,031 | 29.7 | 2,101 | 30.5 | 3,823 | 26.3 |
| ≥85 | 1,564 | 13.2 | 833 | 10.6 | 156 | 5.4 | 50 | 5.2 | 2,066 | 15.2 | 1,080 | 15.7 | 1,446 | 9.9 |
| **Sex** |  |  |  |  |  |  |  |  |  |  |  |  |  |  |
| Male | 7,410 | 62.6 | 4,815 | 61.1 | 1,801 | 62.5 | 598 | 61.7 | 8,178 | 60.4 | 4,132 | 59.9 | 8,730 | 60.0 |
| Female | 4,434 | 37.4 | 3,069 | 38.9 | 1,082 | 37.5 | 371 | 38.3 | 5,374 | 39.7 | 2,766 | 40.1 | 5,830 | 40.0 |
| **Country of birth** |  |  |  |  |  |  |  |  |  |  |  |  |  |  |
| Australia | 8,342 | 70.4 | 5,702 | 72.3 | 1,905 | 66.1 | 635 | 65.5 | 9,649 | 71.2 | 4,956 | 71.9 | 10,427 | 71.6 |
| Other/not known | 3,502 | 29.6 | 2,182 | 27.7 | 978 | 33.9 | 334 | 34.5 | 3,903 | 28.8 | 1,942 | 28.2 | 4,133 | 28.4 |
| **Number of Charlson comorbidities, excluding malignancy** | | | |  |  |  |  |  |  |  |  |  |  |  |
| Nil | 4,263 | 36.0 | 3,523 | 44.7 | 1,313 | 45.5 | 475 | 49.0 | 5,716 | 42.2 | 2,856 | 41.4 | 6,790 | 46.6 |
| 1 comorbidity | 4,553 | 38.4 | 3,178 | 40.3 | 997 | 34.6 | 309 | 31.9 | 5,328 | 39.3 | 2,627 | 38.1 | 5,805 | 39.9 |
| ≥2 comorbidities | 2,101 | 17.7 | 1,183 | 15.0 | 573 | 19.9 | 185 | 19.1 | 2,508 | 18.5 | 1,415 | 20.5 | 1,965 | 13.5 |
| Not known (no hospital admission) | 927 | 7.8 | - | - | - | - | - | - | - | - | - | - | - | - |
| **Other comorbidities** |  |  |  |  |  |  |  |  |  |  |  |  |  |  |
| Depression (yes) | 53 | 0.5 | 32 | 0.4 | 25 | 0.9 | 5 | 0.5 | 70 | 0.5 | 46 | 0.7 | 70 | 0.5 |
| Anxiety-related disorder (yes) | 309 | 2.6 | 161 | 2.0 | 90 | 3.1 | 19 | 2.0 | 292 | 2.2 | 186 | 2.7 | 300 | 2.1 |
| Tobacco use (yes) | 6,391 | 54.0 | 4,333 | 55.0 | 1,656 | 57.4 | 547 | 56.5 | 7,209 | 53.2 | 3,781 | 54.8 | 8,011 | 55.0 |
| Alcohol misuse and dependence (yes) | 232 | 2.0 | 106 | 1.3 | 46 | 1.6 | 16 | 1.7 | 207 | 1.5 | 104 | 1.5 | 190 | 1.3 |
| Drug-related dependence (yes) | 107 | 0.9 | 58 | 0.7 | 21 | 0.7 | 6 | 0.6 | 88 | 0.7 | 40 | 0.6 | 97 | 0.7 |
| **Geographical location of residence** |  |  |  |  |  |  |  |  |  |  |  |  |  |  |
| Urban | 5,903 | 49.8 | 4,966 | 63.0 | 2,073 | 71.9 | 684 | 70.6 | 8,724 | 64.4 | 4,670 | 67.7 | 9,630 | 66.1 |
| Rural | 4,926 | 41.6 | 2,799 | 35.5 | 763 | 26.5 | 259 | 26.7 | 4,585 | 33.8 | 2,19 | 30.9 | 4,759 | 32.7 |
| Not known | 1,015 | 8.6 | 119 | 1.5 | 47 | 1.6 | 26 | 2.7 | 243 | 1.8 | 99 | 1.4 | 171 | 1.2 |
| **Socio-economic status** |  |  |  |  |  |  |  |  |  |  |  |  |  |  |
| Most disadvantaged | 3,160 | 26.7 | 1,658 | 21.0 | 549 | 19.0 | 206 | 21.3 | 3,024 | 22.3 | 1,464 | 21.2 | 2,908 | 20.0 |
| 2 | 3,423 | 28.9 | 2,104 | 26.7 | 685 | 23.8 | 238 | 24.6 | 3,569 | 26.3 | 1,704 | 24.7 | 3,603 | 24.8 |
| 3 | 1,990 | 16.8 | 1,514 | 19.2 | 612 | 21.2 | 167 | 17.2 | 2,513 | 18.5 | 1,279 | 18.5 | 2,811 | 19.3 |
| 4 | 1,142 | 9.6 | 928 | 11.8 | 374 | 13.0 | 119 | 12.3 | 1,594 | 11.8 | 860 | 12.5 | 1,841 | 12.6 |
| Least disadvantaged | 1,115 | 9.4 | 1,561 | 19.8 | 617 | 21.4 | 213 | 22.0 | 2,610 | 19.3 | 1,490 | 21.6 | 3,223 | 22.1 |
| Not known | 1,014 | 5.6 | 119 | 1.5 | 46 | 1.6 | 26 | 2.7 | 242 | 1.8 | 101 | 1.5 | 174 | 1.2 |
| **Resident of aged care** (yes) | 231 | 2.0 | 85 | 1.1 | 14 | 0.5 | # | # | 234 | 1.7 | 248 | 3.6 | 290 | 2.0 |
| **Year of death** |  |  |  |  |  |  |  |  |  |  |  |  |  |  |
| 2014 | 1,876 | 15.8 | 1,255 | 15.9 | 397 | 13.8 | 137 | 14.1 | 2,440 | 18.0 | 1,108 | 16.1 | 2,215 | 15.2 |
| 2015 | 2,002 | 16.9 | 1,394 | 17.7 | 429 | 14.9 | 157 | 16.2 | 2,395 | 17.7 | 1,208 | 17.5 | 2,422 | 16.6 |
| 2016 | 1,883 | 15.9 | 1,340 | 17.0 | 440 | 15.3 | 154 | 15.9 | 2,275 | 16.8 | 1,153 | 16.7 | 2,376 | 16.3 |
| 2017 | 1,908 | 16.1 | 1,343 | 17.0 | 504 | 17.5 | 180 | 18.6 | 2,296 | 16.9 | 1,169 | 17.0 | 2,473 | 17.0 |
| 2018 | 2,022 | 17.1 | 1,261 | 16.0 | 521 | 18.1 | 162 | 16.7 | 2,087 | 15.4 | 1,140 | 16.5 | 2,496 | 17.1 |
| 2019 | 2,153 | 18.2 | 1,291 | 16.4 | 592 | 20.5 | 179 | 18.5 | 2,059 | 15.2 | 1,120 | 16.2 | 2,578 | 17.7 |
| **Hospital type at last admission** |  |  |  |  |  |  |  |  |  |  |  |  |  |  |
| Public | 11,844 | 100.0 | 6,141 | 77.9 | 2,337 | 81.1 | 813 | 83.9 | 9,740 | 71.9 | 4,988 | 72.3 | 11,107 | 76.3 |
| Private | - | - | 1,743 | 22.1 | 546 | 18.9 | 156 | 16.1 | 3,812 | 28.1 | 1,910 | 27.7 | 3,453 | 23.7 |

^1^ Excluding palliative or hospice care. # Cell sizes <5.

**Table S3: Cancer and clinical characteristics of the cancer decedents by potentially burdensome ED and hospital admission care indicators**

|  | **>1 ED visit in last 30 days**  (n=11,844; 14.8%) | | **>1 admission in last 30 days**^1^ (n=7,884; 9.9%) | | **≥1 admission to ICU in last 30 days**  (n=2,883; 3.6%) | | **≥1 mechanical ventilation in last 30 days**  (n=969; 1.2%) | | **Place of death acute care**^1^ (n=13,552; 16.9%) | | **≥14 days in hospital in last 30 days**^1^  (n=6,898; 8.6%) | | **≥3 admissions in last 90 days**^1^  (n=14,560; 18.2%) | |
| --- | --- | --- | --- | --- | --- | --- | --- | --- | --- | --- | --- | --- | --- | --- |
|  | n | % | n | % | n | % | n | % | n | % | n | % | n | % |
| **Age at diagnosis,** median (SD) ^2^ | 69.0 | (13.0) | 68.0 | (13.0) | 67.0 | (13.1) | 66.0 | (13.3) | 71.0 | (13.0) | 71.0 | (12.6) | 68.0 | (12.9) |
| **Time from diagnosis to death** (years), median (SD) ^2^ | 1.8 | (6.9) | 1.9 | (6.7) | 1.9 | (6.8) | 1.8 | (7.4) | 2.0 | (7.1) | 2.0 | (7.1) | 1.8 | (6.5) |
| **History of cancer** (yes) | 3,034 | 25.6 | 2,127 | 27.0 | 787 | 27.3 | 269 | 27.8 | 3,802 | 28.1 | 2,071 | 30.0 | 3,734 | 25.7 |
| **Survival duration** (days) |  |  |  |  |  |  |  |  |  |  |  |  |  |  |
| 31-89 | 1,247 | 10.5 | 765 | 9.7 | 386 | 13.4 | 138 | 14.2 | 1,458 | 10.8 | 826 | 12.0 | 1,759 | 12.1 |
| ≥90 days to <180 | 1,165 | 9.8 | 729 | 9.3 | 301 | 10.4 | 104 | 10.7 | 1,278 | 9.4 | 618 | 9.0 | 1,401 | 9.6 |
| ≥180 | 9,035 | 76.4 | 6,149 | 78.0 | 2,101 | 72.9 | 684 | 70.6 | 10,351 | 76.4 | 5,236 | 75.9 | 11,026 | 75.7 |
| Not known | 397 | 3.4 | 241 | 3.1 | 95 | 3.3 | 43 | 4.4 | 465 | 3.4 | 218 | 3.2 | 374 | 2.6 |
| **Cancer type** |  |  |  |  |  |  |  |  |  |  |  |  |  |  |
| *Head and neck* | 325 | 2.7 | 128 | 1.6 | 71 | 2.5 | 28 | 2.9 | 337 | 2.5 | 143 | 2.1 | 254 | 1.7 |
| *Digestive organs, excl. colorectal* | 2,162 | 18.3 | 1,452 | 18.4 | 378 | 13.1 | 122 | 12.6 | 2,213 | 16.3 | 1,099 | 15.9 | 2,859 | 19.6 |
| *Colorectal* | 1,241 | 10.5 | 874 | 11.1 | 297 | 10.3 | 113 | 11.7 | 1,488 | 11.0 | 705 | 10.2 | 1,651 | 11.3 |
| *Lung* | 2,440 | 20.6 | 1,391 | 17.6 | 511 | 17.7 | 144 | 14.9 | 2,650 | 19.6 | 1,281 | 18.6 | 2,478 | 17.0 |
| *Melanoma of the skin* | 412 | 3.5 | 267 | 3.4 | 98 | 3.4 | 31 | 3.2 | 432 | 3.2 | 258 | 3.7 | 502 | 3.5 |
| *Mesothelial and soft tissue* | 272 | 2.3 | 196 | 2.5 | 70 | 2.4 | 15 | 1.6 | 347 | 2.6 | 161 | 2.3 | 397 | 2.7 |
| *Breast* | 710 | 6.0 | 500 | 6.3 | 146 | 5.1 | 41 | 4.2 | 831 | 6.1 | 366 | 5.3 | 923 | 6.3 |
| *Female genital organs, excl. ovarian* | 209 | 1.8 | 139 | 1.8 | 33 | 1.1 | 14 | 1.4 | 202 | 1.5 | 121 | 1.8 | 251 | 1.7 |
| *Ovarian* | 230 | 1.9 | 183 | 2.3 | 34 | 1.2 | 9 | 0.9 | 251 | 1.9 | 118 | 1.7 | 398 | 2.7 |
| *Prostate* | 789 | 6.7 | 422 | 5.4 | 101 | 3.5 | 21 | 2.2 | 788 | 5.8 | 413 | 6.0 | 779 | 5.4 |
| *Kidney* | 230 | 1.9 | 147 | 1.9 | 56 | 1.9 | 21 | 2.2 | 262 | 1.9 | 143 | 2.1 | 245 | 1.7 |
| *Bladder* | 261 | 2.2 | 152 | 1.9 | 48 | 1.7 | 16 | 1.7 | 293 | 2.2 | 176 | 2.6 | 319 | 2.2 |
| *Neurological* | 194 | 1.6 | 94 | 1.2 | 72 | 2.5 | 30 | 3.1 | 262 | 1.9 | 169 | 2.5 | 273 | 1.9 |
| *Blood and lymphatic system* | 1,560 | 13.2 | 1,387 | 17.6 | 751 | 26.1 | 301 | 31.1 | 2,230 | 16.5 | 1,186 | 17.2 | 2,332 | 16.0 |
| *Unknown primary site* | 561 | 4.7 | 391 | 5.0 | 122 | 4.2 | 26 | 2.7 | 648 | 4.8 | 385 | 5.6 | 589 | 4.1 |
| *Other cancers and other ill-defined sites* | 248 | 2.1 | 161 | 2.0 | 95 | 3.3 | 37 | 3.8 | 318 | 2.4 | 174 | 2.5 | 310 | 2.1 |
| **Degree of cancer spread** |  |  |  |  |  |  |  |  |  |  |  |  |  |  |
| In-situ/localised | 2,141 | 18.1 | 1,303 | 16.5 | 495 | 17.2 | 183 | 18.9 | 2,456 | 18.1 | 1,213 | 17.6 | 2,414 | 16.6 |
| Regionalised | 2,310 | 19.5 | 1,444 | 18.3 | 587 | 20.4 | 201 | 20.7 | 2,727 | 20.1 | 1,344 | 19.5 | 2,788 | 19.2 |
| Metastatic | 3,948 | 33.3 | 2,692 | 34.2 | 743 | 25.8 | 181 | 18.7 | 4,077 | 30.1 | 2,253 | 32.7 | 5,267 | 36.2 |
| Not known | 3,445 | 29.1 | 2,445 | 31.0 | 1,058 | 36.7 | 404 | 41.7 | 4,292 | 31.7 | 2,088 | 30.3 | 4,091 | 28.1 |
| **Place of death** |  |  |  |  |  |  |  |  |  |  |  |  |  |  |
| Home | 950 | 8.0 | 632 | 8.0 | 93 | 3.2 | 8 | 0.8 | 48 | 0.4 | 228 | 3.3 | 1,576 | 10.8 |
| Hospice | 109 | 0.9 | 96 | 1.2 | 18 | 0.6 | # | # | 14 | 0.1 | 78 | 1.1 | 310 | 2.1 |
| Hospital | 7,702 | 65.0 | 5,403 | 68.5 | 2,019 | 70.6 | 706 | 72.9 | 10,738 | 79.2 | 4,916 | 71.3 | 9,085 | 62.4 |
| Residential aged care | 383 | 3.2 | 158 | 2.0 | 23 | 0.8 | # | # | 46 | 0.3 | 258 | 3.7 | 511 | 3.5 |
| Not known | 2,700 | 22.8 | 1,595 | 20.2 | 730 | 25.3 | 246 | 25.4 | 2,706 | 20.0 | 1,418 | 20.6 | 3,078 | 21.1 |

^1^ Excluding palliative care. # Cell sizes <5 or to prevent identification of cell sizes <5.

^2^ Diagnosis age and time from diagnosis to death missing for 3013 people.

**Table S4: Demographic characteristics of the cancer decedents by potentially burdensome chemotherapy or radiotherapy indicators during 2016-2019**

|  | **Last dose chemotherapy in last 7 days**  (n=1,848; 3.5%) | | **Last dose chemotherapy in last 14 days**  (n=3,885; 7.3%) | | **Radiotherapy in last 30 days**  (n=3,072; 5.7%) | |
| --- | --- | --- | --- | --- | --- | --- |
|  | n | % | n | % | n | % |
| **Mean age at death** (SD) | 67.4 | (12.6) | 67.7 | (12.4) | 68.7 | (12.7) |
| **Age group** |  |  |  |  |  |  |
| 20-54 | 281 | 15.2 | 555 | 14.3 | 396 | 12.9 |
| 55-64 | 395 | 21.4 | 809 | 20.8 | 662 | 21.6 |
| 65-74 | 628 | 34.0 | 1,343 | 34.6 | 965 | 31.4 |
| 75-84 | 423 | 22.9 | 935 | 24.1 | 739 | 24.1 |
| ≥85 | 121 | 6.7 | 243 | 6.3 | 310 | 10.1 |
| **Sex** |  |  |  |  |  |  |
| Male | 1,110 | 60.1 | 2,291 | 59.0 | 1,893 | 61.6 |
| Female | 738 | 39.9 | 1,594 | 41.0 | 1,179 | 38.4 |
| **Country of birth** |  |  |  |  |  |  |
| Australia | 1,219 | 66.0 | 2,609 | 67.2 | 2,003 | 65.2 |
| Other/not known | 629 | 34.0 | 1,276 | 32.8 | 1,069 | 34.8 |
| **Number of Charlson comorbidities, excluding malignancy** |  |  |  |  |  |  |
| Nil | 725 | 39.2 | 1,547 | 39.8 | 1,001 | 32.6 |
| 1 comorbidity | 767 | 41.5 | 1,583 | 40.8 | 1,399 | 45.5 |
| ≥2 comorbidities | 272 | 14.7 | 586 | 15.1 | 20 | 0.7 |
| Not known (no hospital admission) | 84 | 4.6 | 169 | 4.4 | 99 | 3.2 |
| **Other comorbidities** |  |  |  |  |  |  |
| Depression (yes) | 10 | 0.5 | 17 | 0.4 | 20 | 0.7 |
| Anxiety-related disorder (yes) | 47 | 2.5 | 104 | 2.7 | 104 | 3.4 |
| Tobacco use (yes) | 981 | 53.1 | 2,068 | 53.2 | 1,815 | 59.1 |
| Alcohol misuse and dependence (yes) | 23 | 1.2 | 47 | 1.2 | 61 | 2.0 |
| Drug-related dependence (yes) | 15 | 0.8 | 36 | 0.9 | 31 | 1.0 |
| **Geographical location of residence** |  |  |  |  |  |  |
| Urban | 1,227 | 66.4 | 2,543 | 65.5 | 2,176 | 70.8 |
| Rural | 528 | 28.6 | 1,144 | 29.5 | 786 | 25.6 |
| Not known | 93 | 5.0 | 198 | 5.1 | 110 | 3.6 |
| **Socio-economic status** |  |  |  |  |  |  |
| Most disadvantaged | 499 | 27.0 | 994 | 25.6 | 770 | 25.1 |
| 2 | 420 | 22.7 | 930 | 23.9 | 733 | 23.9 |
| 3 | 366 | 19.8 | 731 | 18.8 | 548 | 17.8 |
| 4 | 232 | 12.6 | 459 | 11.8 | 457 | 14.9 |
| Least disadvantaged | 239 | 12.9 | 575 | 14.8 | 457 | 14.9 |
| Not known | 92 | 5.0 | 196 | 5.1 | 107 | 3.5 |
| **Resident of aged care** (yes) | 16 | 0.9 | 33 | 0.9 | 89 | 2.9 |
| **Year of death** |  |  |  |  |  |  |
| 2016 | 458 | 24.8 | 920 | 23.7 | 661 | 21.5 |
| 2017 | 446 | 24.1 | 984 | 25.3 | 741 | 24.1 |
| 2018 | 510 | 27.6 | 1,046 | 26.9 | 791 | 25.7 |
| 2019 | 434 | 23.5 | 935 | 24.1 | 879 | 28.6 |

**Table S5: Cancer and clinical characteristics of the cancer decedents by potentially burdensome chemotherapy or radiotherapy indicators during 2016-2019**

|  | **Last dose chemotherapy in last 7 days**  (n=1,848; 3.5%) | | **Last dose chemotherapy in last 14 days**  (n=3,885; 7.3%) | | **Radiotherapy in last 30 days**  (n=3,072; 5.7%) | |
| --- | --- | --- | --- | --- | --- | --- |
|  | n | % | n | % | n | % |
| **Age at diagnosis**^1^**,** median (SD) | 66.0 | (13.0) | 67.0 | (13.0) | 67.0 | (13.1) |
| **Time from diagnosis to death**^1^  (years), median (SD) | 1.7 | (6.4) | 1.7 | (6.4) | 1.3 | (6.4) |
| **History of cancer** (yes) | 435 | 23.5 | 930 | 23.9 | 745 | 24.3 |
| **Survival duration** (days) |  |  |  |  |  |  |
| 31-89 | 216 | 11.7 | 468 | 12.1 | 550 | 17.9 |
| ≥90 days to <180 | 216 | 11.7 | 450 | 11.6 | 351 | 11.4 |
| ≥180 | 1,388 | 75.1 | 2,891 | 74.4 | 2,121 | 69.0 |
| Not known | 28 | 1.5 | 76 | 2.0 | 50 | 1.6 |
| **Cancer type** |  |  |  |  |  |  |
| Head and neck | 55 | 3.0 | 111 | 2.9 | 130 | 4.2 |
| Digestive organs, excl. colorectal | 306 | 16.6 | 635 | 16.3 | 386 | 12.6 |
| Colorectal | 194 | 10.5 | 388 | 10.0 | 177 | 5.8 |
| Lung | 393 | 21.3 | 815 | 21.0 | 1,015 | 33.0 |
| Melanoma of the skin | 56 | 3.0 | 138 | 3.6 | 135 | 4.4 |
| Mesothelial and soft tissue | 51 | 2.8 | 104 | 2.7 | 75 | 2.4 |
| Breast | 160 | 8.7 | 329 | 8.5 | 206 | 6.7 |
| Female genital organs, excl. ovarian | 20 | 1.1 | 49 | 1.3 | 74 | 2.4 |
| Ovarian | 30 | 1.6 | 76 | 2.0 | 15 | 0.5 |
| Prostate | 73 | 4.0 | 149 | 3.8 | 191 | 6.2 |
| Kidney | 22 | 1.2 | 54 | 1.4 | 64 | 2.1 |
| Bladder | 22 | 1.2 | 35 | 0.9 | 71 | 2.3 |
| Neurological | 27 | 1.5 | 55 | 1.4 | 52 | 1.7 |
| Blood and lymphatic system | 324 | 17.5 | 702 | 18.1 | 216 | 7.0 |
| Unknown primary site | 72 | 3.9 | 167 | 4.3 | 173 | 5.6 |
| Other cancers and other ill-defined sites | 43 | 2.3 | 78 | 2.0 | 92 | 3.0 |
| **Degree of cancer spread** |  |  |  |  |  |  |
| In-situ/localised | 277 | 15.0 | 564 | 14.5 | 501 | 16.3 |
| Regionalised | 349 | 18.9 | 739 | 19.0 | 641 | 20.9 |
| Metastatic | 671 | 36.3 | 1,429 | 36.8 | 1,343 | 43.7 |
| Not known | 551 | 29.8 | 1,153 | 29.7 | 587 | 19.1 |
| **Place of death** |  |  |  |  |  |  |
| Home | 211 | 11.4 | 400 | 10.3 | 302 | 9.8 |
| Hospice | # | # | 14 | 0.4 | 29 | 0.9 |
| Hospital | 1,131 | 61.2 | 2,373 | 61.1 | 1,676 | 54.6 |
| Residential aged care | # | # | 46 | 1.2 | 101 | 3.3 |
| Not known | 484 | 26.2 | 1,052 | 27.1 | 964 | 31.4 |

^1^ Diagnosis age and time from diagnosis to death missing for 28, 76 and 50 decedents for last dose chemotherapy in 7 days, in 14 days and radiotherapy in last 30 days, respectively.

# Cell sizes <5 or to prevent identification of cell sizes <5.

**Figure S1a: Predictors of characteristics associated with potentially burdensome care at the end of life by indicator type, 2014-2019**^1,2^


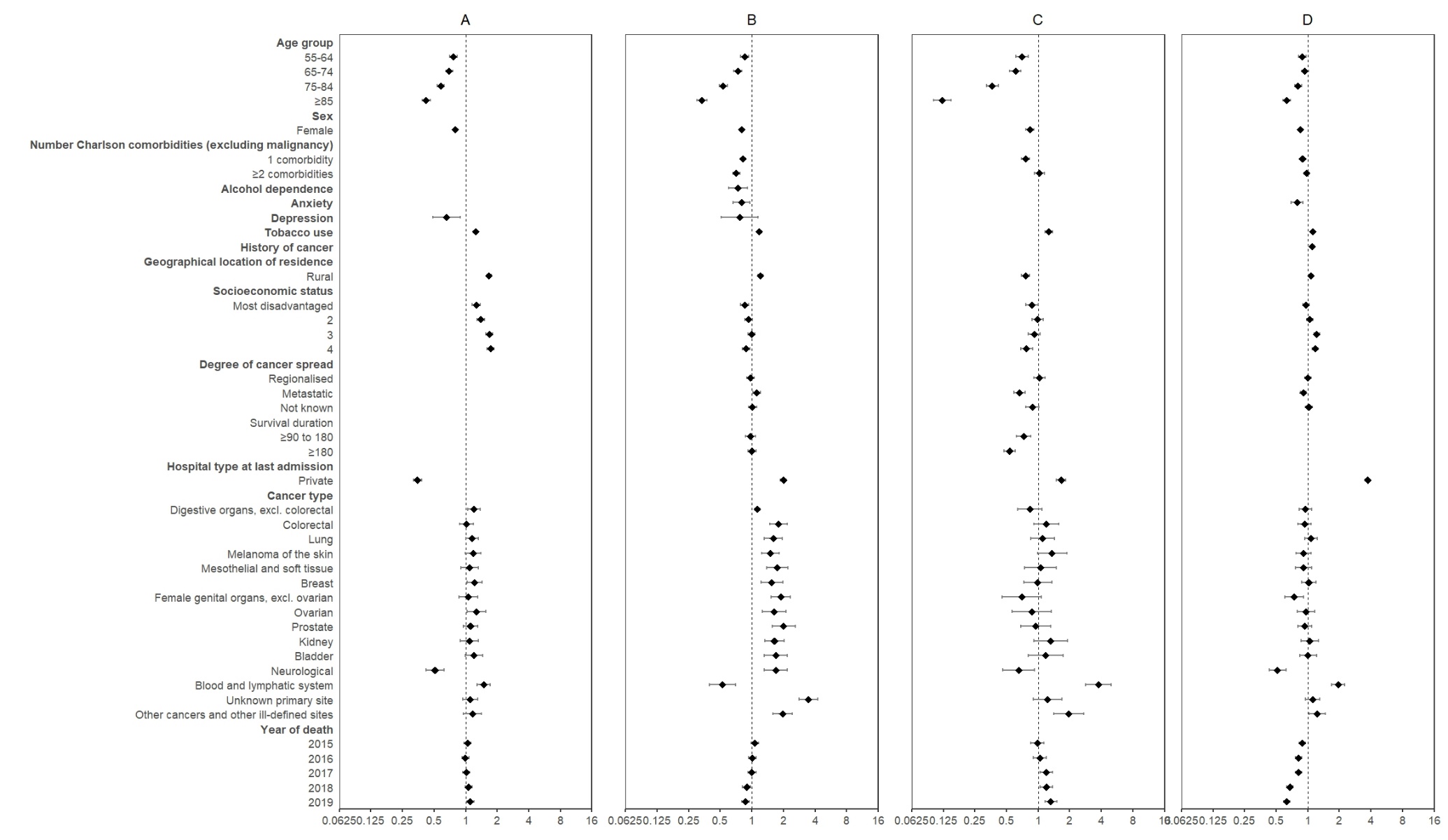


^1^ A=>1 ED visit in last 30 days; B=>1 admission in last 30 days; C=≥1 admission to ICU in last 30 days; D=place of death is acute care; E=≥14 days in hospital in last 30 days; F=≥3 admissions in last 90 days; and G=≥1 mechanical ventilation in last 30 days. All acute care excludes palliative care. Absent OR indicates non-significance in model.

^2^ Reference categories were: 20-54 years, males, nil comorbidities, no history of cancer, urban location, most advantaged socioeconomic status, public hospital, head and neck cancer, and death in 2014.

**Figure S1b: Predictors of characteristics associated with potentially burdensome care at the end of life by indicator type, 2014-2019**^1,2^


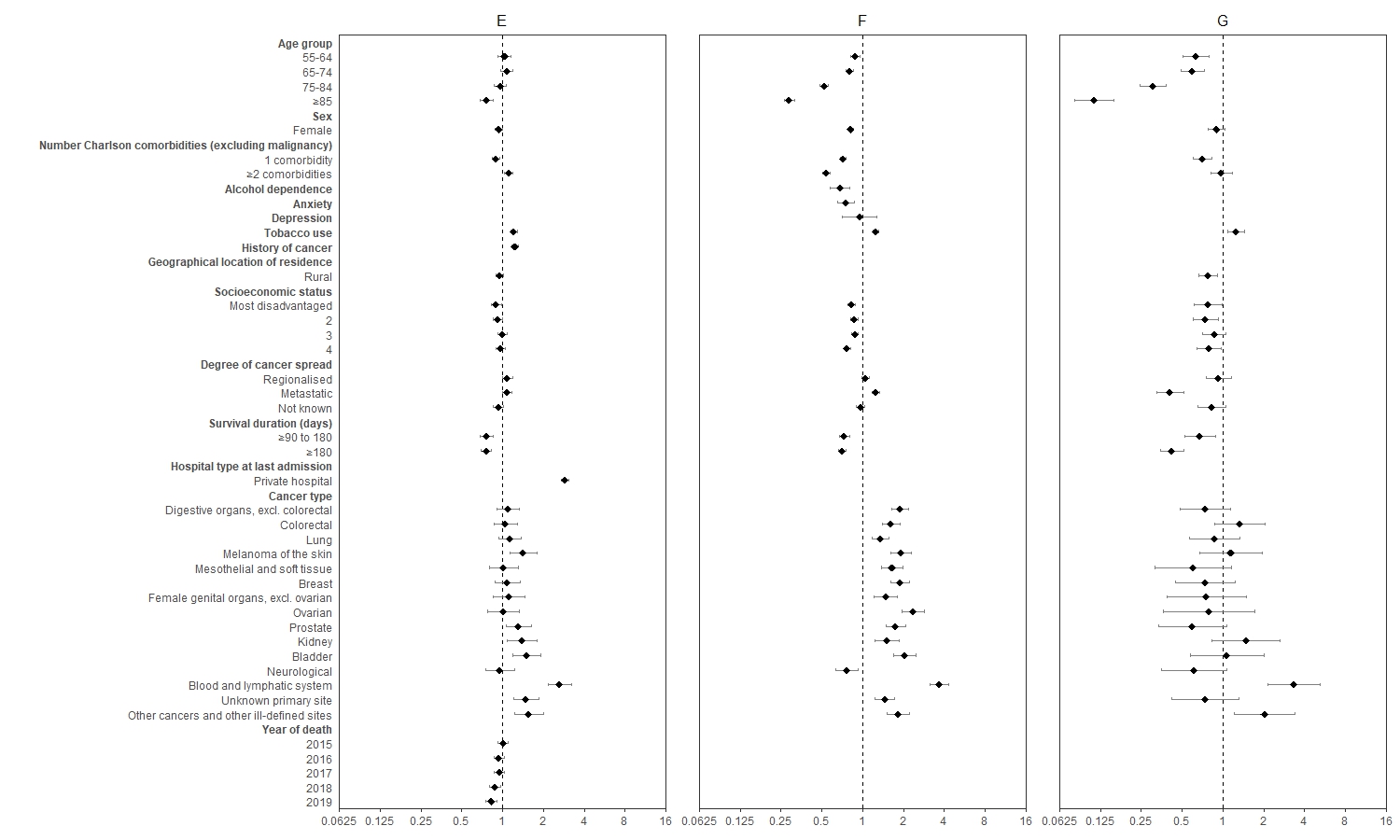


^1^ A=>1 ED visit in last 30 days; B=>1 admission in last 30 days; C=≥1 admission to ICU in last 30 days; D=place of death is acute care; E=≥14 days in hospital in last 30 days; F=≥3 admissions in last 90 days; and G=≥1 mechanical ventilation in last 30 days. All acute care excludes palliative care. Absent OR indicates non-significance in model.

^2^ Reference categories were: 20-54 years, males, nil comorbidities, no history of cancer, urban location, most advantaged socioeconomic status, public hospital, head and neck cancer, and death in 2014.

**Figure S2: Mean number of hospital admissions (a) and ED presentations (b) by month in the last 12 months of life, 2014-2019**

(a) (b)


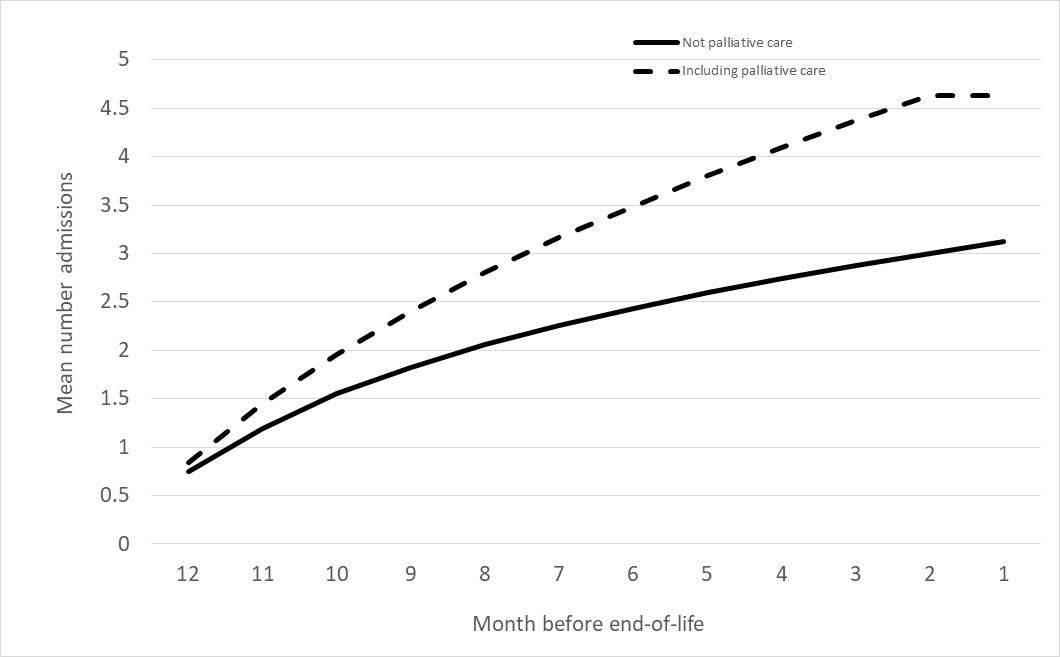

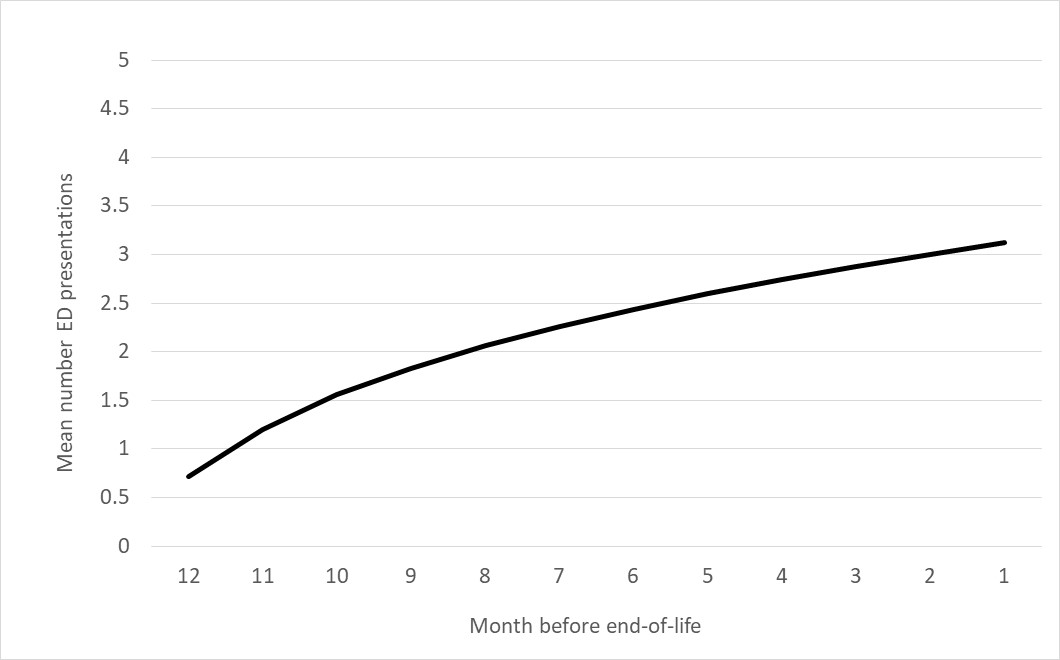


**Figure S3: Mean number of non-admitted patient occasions of service**^1^ **by month in the last 12 months of life, 2016-2019**


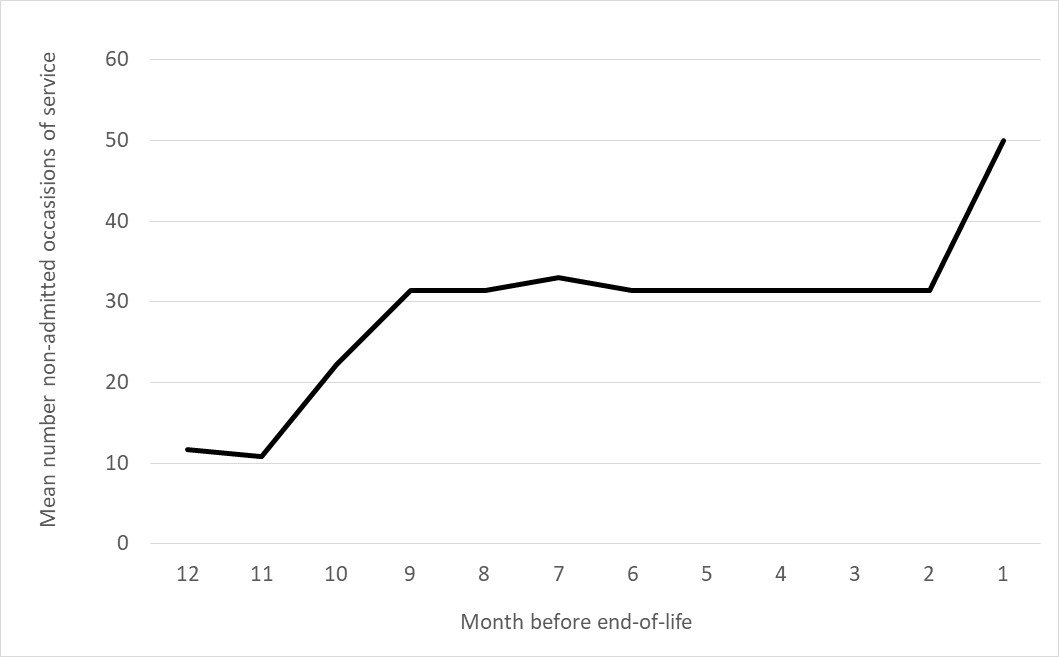


^1^Excludes non-client contacts.
